# Association of spatial proximity to fixed-site syringe services programs with HCV serostatus and injection equipment sharing practices among people who inject drugs in rural New England, United States

**DOI:** 10.1186/s12954-023-00916-5

**Published:** 2024-01-28

**Authors:** Eric Romo, Thomas J. Stopka, Bill M. Jesdale, Bo Wang, Kathleen M. Mazor, Peter D. Friedmann

**Affiliations:** 1 Department of Population and Quantitative Health Sciences, University of Massachusetts Chan Medical School, Worcester, MA USA; 2 Department of Public Health and Community Medicine, Tufts University School of Medicine, Boston, MA USA; 3 Department of Medicine, University of Massachusetts Chan Medical, Worcester, MA USA; 4 Office of Research, University of MA Chan Medical School – Baystate, Springfield, MA USA

**Keywords:** Rural, Syringe services programs, Hepatitis C virus, Spatial proximity, Spatial analysis, Injection drug use, Harm reduction

## Abstract

**Background:**

Hepatitis C virus (HCV) disproportionately affects rural communities, where health services are geographically dispersed. It remains unknown whether proximity to a syringe services program (SSP) is associated with HCV infection among rural people who inject drugs (PWID).

**Methods:**

Data are from a cross-sectional sample of adults who reported injecting drugs in the past 30 days recruited from rural counties in New Hampshire, Vermont, and Massachusetts (2018–2019). We calculated the road network distance between each participant’s address and the nearest fixed-site SSP, categorized as ≤ 1 mile, 1–3 miles, 3–10 miles, and > 10 miles. Staff performed HCV antibody tests and a survey assessed past 30-day injection equipment sharing practices: borrowing used syringes, borrowing other used injection equipment, and backloading. Mixed effects modified Poisson regression estimated prevalence ratios (aPR) and 95% confidence intervals (95% CI). Analyses were also stratified by means of transportation.

**Results:**

Among 330 PWID, 25% lived ≤ 1 mile of the nearest SSP, 17% lived 1–3 miles of an SSP, 12% lived 3–10 miles of an SSP, and 46% lived > 10 miles from an SSP. In multivariable models, compared to PWID who lived within 1 mile of an SSP, those who lived 3 to 10 miles away had a higher prevalence of HCV seropositivity (aPR: 1.25, 95% CI 1.06–1.46), borrowing other used injection equipment (aPR: 1.23, 95% CI 1.04–1.46), and backloading (aPR: 1.48, 95% CI 1.17–1.88). Similar results were observed for PWID living > 10 miles from an SSP: aPR [HCV]: 1.19, 95% CI 1.01–1.40; aPR [borrowing other used equipment]:1.45, 95% CI 1.29–1.63; and aPR [backloading]: 1.59, 95% CI 1.13–2.24. Associations between living 1 to 3 miles of an SSP and each outcome did not reach statistical significance. When stratified by means of transportation, associations between distance to SSP and each outcome (except borrowing other used injection equipment) were only observed among PWID who traveled by other means (versus traveled by automobile).

**Conclusions:**

Among PWID in rural New England, living farther from a fixed-site SSP was associated with a higher prevalence of HCV seropositivity, borrowing other used injection equipment, and backloading, reinforcing the need to increase SSP accessibility in rural areas. Means of transportation may modify this relationship.

**Supplementary Information:**

The online version contains supplementary material available at 10.1186/s12954-023-00916-5.

## Introduction

The United States is amid an ongoing epidemic of hepatitis C virus (HCV) infections. Between 2010 and 2020, the number of annual new HCV infections in the United States increased by over 4.5-fold [1], an increase largely attributed to a rise in injection drug use and related injection practices shown to be associated with acquiring HCV. These include sharing syringes, sharing other injection equipment (e.g., cotton filters, cookers), and backloading (injecting drugs that someone else prepared, mixed, or divided with a used syringe) [2–4]. These new HCV infections have occurred disproportionately among young people who inject drugs (PWID) living in rural areas [5, 6]. From 2006 to 2012, the incidence of HCV among young adults in rural counties was nearly twice that of young adults in urban counties [7].

Syringe services programs (SSP) serve as important interventions to reduce transmission of bloodborne pathogens. SSPs provide PWID with sterile syringes and other injection equipment (e.g., cotton filters, cookers) and often provide a range of other services, including infectious disease testing and referral to substance use disorder treatment. Most SSPs are fixed-site, but some operate as mobile units or offer on-call delivery services [8]. It is well established that obtaining syringes from SSPs reduces injection equipment sharing practices and HIV transmission among PWID [9, 10]. However, a recent review of reviews concluded that there was only tentative evidence that SSPs are effective at reducing HCV, with the strongest evidence coming from European studies [9, 10]. Given HCV is more transmissible through percutaneous blood exposure than HIV, is unclear whether SSPs sufficiently reduce injection equipment sharing practices to a level that lowers the risk of HCV transmission. Despite the disproportionate HCV burden among rural PWID, the impact of SSPs on HCV risk in rural settings is understudied.

SSPs have been increasingly conceptualized as structural interventions that alter the environment in ways that influence the risk of drug-related harms. SSPs alter the physical environment by increasing the number of sterile syringes circulating in a geographic space. Research has shown that this approach lowers the prevalence of HIV in circulating syringes, thus decreasing the risk of HIV infection among PWID [11]. By this same mechanism, sterile syringe sources could potentially decrease the risk of HCV, not just for PWID who directly utilize these services but for anyone in physical proximity to a fixed-site SSP. Previous studies have shown that spatial proximity to SSPs in urban locations is associated with a reduction in injection equipment sharing practices among PWID [12–16]. However, to our knowledge, no study has evaluated the association between spatial proximity to fixed-site SSPs and HCV risk among PWID, especially among rural PWID. Understanding the impact of spatial proximity to SSPs may be especially important for PWID in rural communities, where populations are more geographically dispersed and spatial access to services is a more prominent issue than in urban communities.

Using data from the Drug Injection Surveillance and Care Enhancement for Rural Northern New England (DISCERNNE) study, we examined the association between spatial proximity to the nearest fixed-site SSP and HCV seroprevalence. A secondary study goal was to examine the relationship between spatial proximity to the nearest fixed-site SSP and three injection equipment sharing practices: borrowing used syringes, borrowing other used injection equipment, and backloading.

## Methods

### Data source

The study described here was conducted in the context of the DISCERNNE study, a multi-site, mixed-methods cross-sectional study of people who use drugs in rural Northern New England, United States. The study was conducted in 11 rural counties in New Hampshire (NH), Vermont (VT), and Massachusetts (MA) located along the Connecticut River Valley. Overall, DISCERNNE aimed to characterize the risk environment and epidemiology of overdose and injection-mediated infectious diseases in a rural setting. Further details and findings from DISCERNNE have been reported elsewhere [17–20].

### Study participants

**Fig. 1:**
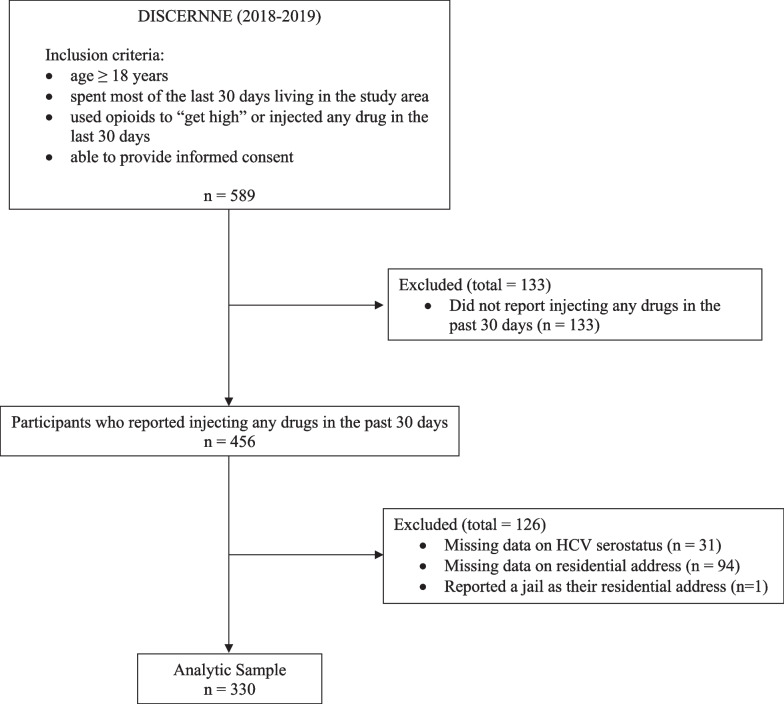
Flow diagram of study sample

Participants were eligible for the DISCERNNE study if they: (1) were ≥ 18 years old, (2) spent most of the last 30 days living in the study area, (3) used opioids to “get high” or injected any drug in the last 30 days, and (4) were able to provide informed consent. Study staff recruited participants at 11 study sites that were chosen after consulting local public health officials, service providers, and harm reduction experts. Four of the 11 study sites were co-located with existing fixed-site SSPs. Participants were recruited using respondent-driven sampling (RDS), a chain-referral sampling method for reaching and recruiting hidden populations [21]. Two to six RDS “seeds” were recruited at each study site. Staff recruited seeds through street outreach and at harm reduction agencies. Upon completion of the study survey, RDS seeds were given three uniquely coded coupons to recruit their eligible peers into the study. Participants who had all three coupons redeemed were offered additional coupons. Study participants continued referring their peers through multiple waves of recruitment until the desired sample size was reached. Participants received $10 USD for each referred peer who redeemed a coupon and completed the study survey. Study recruitment occurred between May 2018 and October 2019. A total of 589 participants were enrolled and completed the study survey. However, only participants who reported injecting any drug in the last 30 days (*n* = 456) were considered for this analysis. Of these 456 PWID participants, 31 (7%) were excluded for having incomplete information on HCV serostatus, 94 (21%) for incomplete information on residential address, and 1 (0.2%) for reporting a jail as their residential address, resulting in a final analytic sample of 330 PWID (Fig. 1). The Baystate Health Institutional Review Board approved the study protocol. A U.S. Federal Certificate of Confidentiality issued by the National Institutes of Health protects the study data from subpoena.

### Data collection

Participant-level data were collected through a 90-min Audio Computer-Assisted Self-Interview (ACASI) that participants completed on a touch-screen laptop. ACASI allows participants to confidentially answer sensitive questions and increases accurate reporting of substance use and other sensitive behaviors [22]. The ACASI collected information on participants’ sociodemographic characteristics, lifetime and recent substance use history, recent injection and sexual behaviors, overdose history, lifetime and recent substance use treatment, healthcare utilization, and infectious disease history. Participants were tested for HCV antibodies using the OraQuick HCV rapid antibody test (sensitivity = 0.99, specificity = 1.00) [23]. Participants received $20 USD for completing the ACASI and $20 USD for infectious disease testing.

### Measures

#### Spatial proximity to SSP

Five fixed-site SSPs were operating in the study area during the study period (4 in VT, 1 in MA). All SSPs were operated by regional non-profit organizations, four of which were AIDS service organizations. All fixed-site SSPs offered similar services, including sterile syringes and other injection equipment (e.g., cotton filters, cookers, sterile water), HIV/HCV testing, naloxone kits, harm reduction education, and case management services. Four of the five SSPs offered some form of mobile SSP services. However, when participants were asked “How close is the nearest syringe or needle exchange program to where you live?”, only 1 participant responded with “there is a mobile program that comes near where I live”, suggesting that very few PWID in our sample were even aware of mobile SSP programs in the area.

Spatial proximity to the nearest fixed-site SSP was defined as the road network distance between a participant’s residential street address and the nearest fixed-site SSP. Participants were asked in the ACASI for the street address, town name, and postal code of their residence, defined as where they had slept most in the past 30 days. The addresses of all fixed-site SSPs operating in the study area during the study period were compiled from departments of public health websites, state epidemiologists, and other publicly available databases. Participant addresses and SSP sites were geocoded to the street address level using 2019 U.S. Census TIGER/Lines shapefiles in ArcMap 10.7.1 (Esri, Redlands, CA, USA). Using the origin–destination cost matrix analysis tool in ArcGIS Pro, a geographic information system (GIS), we calculated the shortest road network distance between each participant’s residence and the nearest fixed-site SSP. Road network distance offers a travel distance that is more accurate than the Euclidean distance between two points [24].

Rather than treat road network distance as a continuous variable, we felt that it was more appropriate to categorize road network distance into categories that were conceptually meaningful for our study area. We categorized road network distance into four conceptually meaningful categories: ≤ 1 mile (≤ 1.6 km, walking distance), 1–3 miles (1.6–4.8 km, short driving distance), 3–10 miles (4.8–16.1 km, medium driving distance), and > 10 miles (> 16.1 km, long driving distance). We selected these cutoffs based on previous research and the population density of our rural study area. Past research has suggested 1 mile as a reasonable distance people are willing to walk to reach community services [25, 26], and past research in an urban setting suggests that 1 mile is the distance PWID are willing to walk to a fixed-site SSP [27]. Studies in rural Canada evaluating geographic access to pharmacies have used a cutoff of 5 km (~ 3 miles) to define short driving distance [28, 29]. In our rural study area, a travel distance of 3 miles corresponds to in-town travel for most participants. A cutoff of 10 miles has frequently been used in studies of rural geographic access to healthcare [30–32], and there is research that suggests 10 miles may be the limit of a convenient driving distance for rural drivers [33]. In our study area, a travel distance greater than 10 miles corresponds to out-of-town travel for most participants.

#### HCV serostatus and injection equipment sharing practices

Our primary study outcome was HCV serostatus. Participants were considered HCV seropositive if they had a positive rapid HCV antibody test. Secondary study outcomes included three past 30-day injection equipment sharing practices: borrowing used syringes (using a syringe known to have been used by someone else), borrowing other used injection equipment (using a cotton, cooker, or water for rinsing/mixing known to have been used by someone else), and backloading (injecting drugs that someone else prepared, mixed, or divided with a used syringe). These practices are known risk factors for HCV and may serve as mediators of the association between distance to the nearest fixed-site SSP and HCV serostatus.

#### Potential confounding variables

We selected confounders for adjustment based on the disjunctive cause criterion. Under this criterion, sufficient control for confounding can be achieved by adjusting for variables that cause the exposure, outcome, or both, and avoiding adjusting for known instrumental variables [34, 35]. Based on the literature [36–42], we identified several sociodemographic and injection-related variables that satisfied this criterion. Sociodemographic variables included age (years), gender (men, women), race (White, non-White), sexual orientation (heterosexual, bisexual/homosexual/other), incarceration within the past 6 months (yes/no) and homelessness within the past 6 months (yes/no). Injection-related variables included years injecting (continuous); injection frequency in the past 30 days (at least once a day, less than daily); injecting multiple times per sitting within the past 30 days (yes/no); injecting heroin (yes/no), cocaine (yes/no), methamphetamine (yes/no), or simultaneous injection of an opioid and cocaine or an opioid and methamphetamine (i.e., speedball or goofball, respectively) (yes/no) within the past 30 days; and receiving medications for opioid use disorder [MOUD] (ever, never).

#### Consideration of means of transportation

An individual’s means of transportation influences how they experience a given travel distance. For example, someone traveling a given distance in an automobile will incur lower travel “costs” (in terms of time and physical exertion) compared to someone traveling the same distance on a bicycle. Means of transportation may be especially salient in rural areas, where, compared to urban areas, individuals often must travel longer distances to reach healthcare and substance use treatment services [43, 44]. To explore the potential role of means of transportation as an effect modifier, we evaluated whether the association between distance to the nearest fixed-site SSP and the outcomes of interest differed by means of transportation.

We assessed participants’ means of transportation with the question: “If you need to go somewhere more than a mile away from home, how do you usually get there?” We dichotomized responses into travel by automobile (“drive my own car or truck”, “ride with someone else”, or “borrow someone else’s car or truck”) versus travel by other means (“walk”, “bus”, “bicycle”, or “other”). When defining these two categories, our goal was to group together methods of transportation that afforded relatively similar levels of freedom and convenience to travel to a fixed-site SSP. It was our opinion that having access to a personal vehicle in any capacity offered greater freedom and convenience to travel to a fixed-site SSP compared to relying on some other means of transportation (e.g., walking, taking the bus).

### Statistical analysis

The statistical analysis consisted of three steps: (1) describing the overall sample and evaluating the association of distance to the nearest fixed-site SSP with HCV serostatus and injection equipment sharing practices; (2) performing a stratified analysis by means of transportation; and (3) performing a sensitivity analysis by restricting our primary analysis to PWID who self-reported primarily obtaining syringes in the past 30 days from a source other than an SSP. First, we compared participant characteristics, HCV serostatus, and injection equipment sharing practices across the four categories of road network distance to the nearest fixed-site SSP (≤ 1 mile, 1–3 miles, 3–10 miles, > 10 miles) using chi-square tests and ANOVA for discrete and continuous variables, respectively. We used multivariable mixed effects modified Poisson regressions to model the relationship between road network distance to the nearest fixed-site SSP and our primary and secondary outcomes. Mixed effects models accounted for the lack of independence among participants within study sites with a random intercept for study site. Modified Poisson models allow for the direct estimation of prevalence ratios for common binary outcomes [45, 46]. We adjusted for all potential confounders discussed previously in our final multivariable models. RDS weights were not applied to regression models, as (1) such methods were developed for prevalence estimate and (2) RDS weighting has been shown to increase bias and type 1 error in association studies [47].

Second, we repeated the analysis in the first step and stratified by means of transportation to explore whether means of transportation plays a role in modifying any association between distance to the nearest fixed-site SSP and the primary and secondary outcomes. We collapsed distance to the nearest fixed-site SSP from four to two categories (≤ 3 miles, > 3 miles) [≤ 4.8 km, > 4.8 km] to avoid small cell sizes (*n* < 10).

In the third step of the analysis, we performed a sensitivity analysis. We repeated the analysis in the first step but restricted our sample to PWID who reported primarily obtaining syringes in the past 30 days from a source other than an SSP. These other syringe sources included both sterile sources (e.g., pharmacies) and potentially unsterile sources (e.g., friend, drug dealer). The goal was to explore whether any associations between distance to the nearest fixed-site SSP and the outcomes of interest were present among PWID who did not directly use SSPs themselves. If so, this could suggest that the associations between distance to the nearest fixed-site SSP and the outcomes are not fully explained by mediation through direct SSP use.

We assessed for the presence of multicollinearity in all multivariable models using variance inflation factors (VIFs) and did not find it to be a problem, as no VIF exceeded 2.0. All analyses were performed using Stata version 17.0 (Stata Corp LP, College Station, TX, USA).

## Results

### Participant sociodemographic and injection-related characteristics

Participants in the analytic sample were predominantly men (58%), White (91%), injected heroin at least once in the past 30 days (86%), and reported injecting drugs at least daily (57%) in the past 30 days. More than half (52%) reported homelessness in the past 6 months (Table 1). The median age of the participants was 34 years (Q1–Q3: 28–41), and the median number of years of drug injection was 9 years (Q1–Q3: 4–16). Almost three-quarters of participants (72%) tested positive for HCV antibodies. Nearly one-half (45%) reported borrowing used syringes, while over half (54%) reported borrowing other used injection equipment, and almost half (45%) reported backloading in the past 30 days.

**Table 1:** Participant characteristics by road network distance to the nearest fixed-site SSP

Characteristic	≤ 1 mile (*n* = 84)	1—3 miles (*n* = 56)	3—10 miles (*n* = 39)	> 10 miles (*n* = 151)	Test statistic (*p*-value)
*Sociodemographics—no. (%)*					
Gender: women	39 (46)	23 (41)	13 (33)	62 (41)	*χ*^2^ = 5.0 (0.54)
Age (years)—median (Q1–Q3)	39 (31–47)	34 (29–40)	35 (27–45)	32 (28–39)	*F* = 4.5 (0.004)
White race	72 (86)	54 (96)	34 (87)	141 (93)	*χ*^2^ = 6.8 (0.08)
Sexual orientation: bisexual/homosexual/other^a^	13 (15)	6 (11)	5 (13)	27 (18)	*χ*^2^ = 3.1 (0.80)
High school education or higher	62 (74)	42 (75)	29 (74)	110 (73)	*χ*^2^ = 0.1 (0.99)
Experienced homelessness (past 6 months)	55 (65)	19 (34)	17 (44)	79 (52)	*χ*^2^ = 15.8 (0.02)
*Criminal justice involvement—no. (%)*					
Incarcerated (past 6 months)	24 (29)	11 (20)	9 (23)	62 (41)	*χ*^2^ = 11.5 (0.01)
*Injection drug use—no. (%)*					
Years of injection—median (Q1–Q3)	12 (5–20)	8 (5–17)	9 (3–15)	8 (4–13)	*F* = 2.2 (0.09)
Inject at least daily (past 30 days)	50 (60)	38 (68)	25 (64)	74 (49)	*χ*^2^ = 10.3 (0.11)
Inject multiple times per sitting (past 30 days)	60 (71)	40 (71)	31 (79)	107 (71)	*χ*^2^ = 7.3 (0.29)
Inject heroin (past 30 days)	77 (92)	50 (89)	37 (95)	119 (79)	*χ*^2^ = 13.0 (0.04)
Inject cocaine (past 30 days)	44 (52)	22 (39)	21 (54)	73 (48)	*χ*^2^ = 5.2 (0.52)
Inject methamphetamine (past 30 days)	19 (23)	14 (25)	9 (23)	38 (25)	*χ*^2^ = 0.6 (1.00)
Inject speedball or goofball (past 30 days)	32 (38)	16 (29)	16 (41)	41 (27)	*χ*^2^ = 8.9 (0.18)
*Primary syringe source (past 30 days)—no. (%)*					
SSP (vs. other source^b^)	27 (32)	15 (27)	11 (28)	9 (6)	*χ*^2^ = 33.9 (< 0.001)
*Addiction treatment—no. (%)*					
Ever received MOUD	64 (76)	49 (88)	26 (67)	113 (75)	*χ*^2^ = 8.6 (0.20)
*Infectious disease—no. (%)*					
HCV seropositive	56 (67)	39 (70)	30 (77)	111 (74)	*χ*^2^ = 1.9 (0.59)
*Injection equipment sharing (past 30 days)—no. (%)*					
Borrowing used syringes	34 (40)	18 (32)	12 (31)	83 (55)	*χ*^2^ = 22.4 (0.001)
Borrowing other used injection equipment	42 (50)	21 (38)	21 (54)	95 (63)	*χ*^2^ = 21.8 (0.001)
Backloading	31 (37)	20 (36)	17 (44)	79 (52)	*χ*^2^ = 16.6 (0.01)
*Means of transportation when traveling > 1 mile—no. (%)*					
Travel by automobile^c^	33 (39)	25 (45)	21 (54)	74 (49)	χ^2^ = 3.1 (0.38)
Travel by other means^d^	51 (61)	31 (55)	18 (46)	77 (51)	

SSP, Syringe services program; MOUD, Medications for opioid use disorder; HCV, Hepatitis C virus

Chi-square tests were used for categorical variables, and ANOVA was used for continuous variables

^a^Most PWID in the “bisexual/homosexual/other” category identified as bisexual (88.4%)

^b^Other syringe sources include pharmacy; secondary syringe exchange; friend or acquaintance; drug dealer or street syringe seller; spouse, partner, or relative; or they found their syringes

^c^Travel by automobile = drive their own car, ride with someone else, or borrow someone else’s car or truck

^d^Travel by other means = walk, bus, bicycle, ATV/snowmobile, or other means

With respect to road network distance to the nearest fixed-site SSP, 25% lived within 1 mile of a fixed-site SSP, 17% lived between 1 and 3 miles of a fixed-site SSP, 12% lived between 3 and 10 miles of a fixed-site SSP, and the remaining 46% lived greater than 10 miles from a fixed-site SSP. Those who lived within 1 mile of a fixed-site SSP were older, on average, than those who lived farther distances from a fixed-site SSP (Table 1) and were more likely to report experiencing homelessness in the past 6 months. PWID who lived greater than 10 miles from a fixed-site SSP were the least likely to report past-month injection of heroin (78%) but were the most likely to be recently incarcerated (41%). Those living greater than 10 miles from a fixed-site SSP were the most likely to report borrowing used syringes, borrowing other used injection equipment, and backloading.

### Association of distance to nearest fixed-site SSP with HCV and injection equipment sharing practices

Compared with those living within 1 mile of a fixed-site SSP, those who lived 3 to 10 miles away had a 25% higher HCV seroprevalence (aPR: 1.25, 95% CI 1.06–1.46) and those who lived greater than 10 miles away had a 19% higher HCV seroprevalence (aPR: 1.19, 95% CI 1.01–1.40) after adjusting for sociodemographic and injection-related factors (Table 2). We did not observe a statistically significant association between living 1 to 3 miles from the nearest fixed-site SSP and HCV seropositivity (aPR: 1.12, 95% CI 0.94–1.33).

**Table 2:** Associations between road network distance to nearest fixed-site SSP and HCV seropositivity

Distance to nearest fixed-site SSP	Crude PR (95% CI)	Adjusted PR^a^ (95% CI)
≤ 1 mile	reference	reference
1–3 miles	1.04 (0.94–1.16)	1.12 (0.94–1.33)
3–10 miles	1.15 (1.03–1.29)	1.25 (1.06–1.46)
> 10 miles	1.10 (0.88–1.38)	1.19 (1.01–1.40)

PR, Prevalence ratio; CI, Confidence interval

^a^Adjusted for age, gender, race, sexual orientation, incarceration, homelessness, years of injection, injection frequency, inject multiple times per sitting, inject heroin, inject cocaine, inject methamphetamine, inject speedball/goofball, ever received medication for opioid use disorderfor sociodemographic factors

In the final multivariable adjusted models for injection equipment sharing practices, compared with those who lived within 1 mile of a fixed-site SSP, those living 3 to 10 miles from the nearest fixed-site SSPs were more likely to report borrowing other used injection equipment (aPR: 1.23; 95% CI 1.04–1.46) and backloading (aPR: 1.48; 95% CI 1.17–1.88) (Table 3). Similarly, compared with those who lived within 1 mile of a fixed-site SSP, those who lived greater than 10 miles from the nearest fixed-site SSP were more likely to report borrowing other used injection equipment (aPR: 1.45; 95% CI 1.29–1.63) and backloading (aPR: 1.59; 95% CI 1.13–2.24) (Table 3). After adjusting for sociodemographic and injection-related factors, we did not observe a statistically significant association between living greater than 10 miles from the nearest fixed-site SSP and borrowing used syringes (aPR: 1.25; 95% CI 0.57–2.79).

**Table 3:** Associations between distance to nearest fixed-site SSP and injection equipment sharing practices

Outcome	Distance to nearest fixed-site SSP	Crude PR (95% CI)	Adjusted PR^a^ (95% CI)
Borrowing used syringes	≤ 1 mile	reference	reference
	1–3 miles	0.79 (0.49–1.28)	0.91 (0.59–1.41)
	3–10 miles	0.75 (0.48–1.16)	0.85 (0.59–1.23)
	> 10 miles	1.11 (0.63–1.96)	1.25 (0.57–2.79)
Borrowing other used injection equipment	≤ 1 mile	reference	reference
	1–3 miles	0.77 (0.63–0.94)	0.91 (0.70–1.17)
	3–10 miles	1.14 (0.94–1.38)	1.23 (1.04–1.46)
	> 10 miles	1.36 (1.12–1.67)	1.45 (1.29–1.63)
Backloading	≤ 1 mile	reference	reference
	1–3 miles	0.99 (0.69–1.42)	1.30 (0.98–1.71)
	3–10 miles	1.20 (0.81–1.78)	1.48 (1.17–1.88)
	> 10 miles	1.41 (0.58–3.43)	1.59 (1.13–2.24)

PR, Prevalence ratio; CI, Confidence interval

^a^Adjusted for age, gender, race, sexual orientation, incarceration, homelessness, years of injection, injection frequency, inject multiple times per sitting, inject heroin, inject cocaine, inject methamphetamine, inject speedball/goofball, ever received medication for opioid use disorder

### Stratified analysis by means of transportation

Forty-six percent of participants reported usually traveling by automobile when going more than 1 mile from home. This included riding with someone else (63%), driving one’s own car/truck (31%), and borrowing someone else’s car/truck (5%). The other 53% reported usually traveling by other means, most commonly walking (53%), traveling by bus (25%), or biking (13%). Among the overall sample, associations between the two-category variable for distance to the nearest fixed-site SSP (> 3 miles vs. ≤ 3 miles) and each outcome were largely consistent with the associations observed when using the original four-category distance variable; compared to living within 3 miles of a fixed-site SSP, living greater than 3 miles away was associated with a higher HCV seroprevalence (aPR: 1.15; 95% CI 1.00–1.32) and a higher prevalence of borrowing other used injection equipment (aPR: 1.45; 95% CI 1.26–1.67) and backloading (1.41; 95% CI 0.97–2.05), although this final association did not reach the threshold for statistical significance (Table 4).

**Table 4:** Associations between distance to nearest fixed-site SSP and primary and secondary outcomes by means of transportation

Outcome	Distance to nearest fixed-site SSP	**Overall** Adjusted PR^c^ (95% CI)	**Travel by auto**^**a**^ Adjusted PR^c^ (95% CI)	**Travel by other means**^**b**^ Adjusted PR^c^ (95% CI)
HCV seropositive	≤ 3 miles	reference	reference	reference
	> 3 miles	1.15 (1.00–1.32)	1.07 (0.81–1.41)	1.23 (1.03–1.47)
Borrowing used syringes	≤ 3 miles	reference	reference	reference
	> 3 miles	1.09 (0.71–1.68)	1.04 (0.69–1.57)	1.69 (1.20–2.38)
Borrowing other used injection equipment	≤ 3 miles	reference	reference	reference
	> 3 miles	1.45 (1.26–1.67)	1.58 (1.17–2.14)	1.36 (1.02–1.80)
Backloading	≤ 3 miles	reference	reference	reference
	> 3 miles	1.41 (0.97–2.05)	1.11 (0.73–1.69)	1.76 (1.11–2.79)

PR = Prevalence ratio; CI = Confidence interval

^a^Travel by automobile = drive their own car, ride with someone else, or borrow someone else’s car or truck (*n* = 153, 46% of total sample)

^b^Travel by other means = walk, bus, bicycle, ATV/snowmobile, or other means (*n* = 177, 54% of total sample)

^c^Adjusted for age, gender, race, sexual orientation, incarceration, homelessness, years of injection, injection frequency, inject multiple times per sitting, inject heroin, inject cocaine, inject meth, inject speedball/goofball, ever received medication for opioid use disorder

When stratified by means of transportation, living greater than 3 miles from the nearest fixed-site SSP (versus living within 3 miles) was associated with a higher HCV seroprevalence (aPR: 1.23; 95% CI 1.03–1.47) and a higher prevalence of borrowing used syringes (aPR: 1.69; 95% CI 1.20–2.38) and backloading (aPR: 1.76; 95% CI 1.11–2.79) among PWID who traveled by means other than an automobile, but not among PWID who traveled by automobile (Table 4). In contrast, living greater than 3 miles (versus living within 3 miles) from the nearest fixed-site SSP was associated with a higher prevalence of borrowing other used injection equipment among both those who traveled by automobile and those who traveled by other means, with the association actually being stronger in magnitude among PWID who traveled by automobile (aPR: 1.58; 95% CI 1.17–2.14 vs. aPR: 1.36; 95% CI 1.02–1.80).

### Sensitivity analysis

We performed a sensitivity analysis by repeating our primary analysis but restricted the sample to PWID who reported primarily obtaining syringes in the past 30 days from a source other than an SSP (*n* = 242). Among this restricted sample, living a farther distance from a fixed-site SSP remained associated with a higher HCV seroprevalence and a higher prevalence of injection equipment sharing practices (Additional file 1: Table S1). Effect estimates were slightly different in the restricted sample, but there was no difference in the overall conclusions.

## Discussion

Among a sample of PWID living in rural northern New England, we observed that living a farther distance from a fixed-site SSP was associated with a higher prevalence of HCV seropositivity, borrowing other used injection equipment, and backloading. When stratified by means of transportation, positive associations between distance to the nearest fixed-site SSP (> 3 miles vs. ≤ 3 miles) and each outcome (except borrowing other used injection equipment) were only observed among PWID who usually traveled by means other than an automobile.

Compared to PWID living within a mile of the nearest fixed-site SSP, those living farther away had a higher HCV seroprevalence. Although previous studies have examined the relationship between distance to a fixed-site SSP and injection equipment sharing practices, our study is the first, to our knowledge, to evaluate the association between distance to SSP and HCV seroprevalence. Our results suggest that rural PWID living closer to a fixed-site SSP might have been less likely to have acquired HCV. Our results could indicate that living closer to a fixed-site SSP may lower the risk of HCV infection among PWID in rural settings; however, given these data are cross-sectional and HCV seroprevalence does not allow us to differentiate between incident and prevalent infections, it is possible that PWID were infected with HCV before they began living at the residential addresses we used to calculate distance to fixed-site SSP.

When stratified by means of transportation, associations between distance to fixed-site SSP (> 3 miles vs. ≤ 3 miles) and HCV seroprevalence were observed only among those who traveled by means other than an automobile. This suggests, at least in our rural study setting, that a PWID’s means of transportation may modify the relationship between distance to fixed-site SSP and HCV seroprevalence. It makes intuitive sense that living a given distance from a fixed-site SSP while having access to an automobile would be a different experience from living the same distance from an SSP while only having access to a bicycle.

As has been seen in previous urban studies [12–16], we observed that living a greater distance from a fixed-site SSP was associated with a higher prevalence of borrowing used syringes, borrowing other used injection equipment, and backloading. Both living 3 to 10 miles and living greater than 10 miles from a fixed-site SSP were associated with a higher prevalence of borrowing other used injection equipment and backloading. However, the effect estimates were larger in magnitude for those living greater than 10 miles from a fixed-site SSP, suggesting a possible dose–response relationship. If we believe living farther from a fixed-site SSP increases the risk of injection equipment sharing practices by decreasing the likelihood of utilizing a fixed-site SSP, one might have expected the association of distance to fixed-site SSP with borrowing used syringes to be similar or even stronger in magnitude than the associations with borrowing other used injection equipment and backloading. However, only living greater than 10 miles from a fixed-site SSP was associated with a higher prevalence of borrowing used syringes, and this association was not statistically significant. This might be explained by the fact that some PWID who lived farther away from a fixed-site SSP were still able to obtain clean syringes from a local pharmacy. The likelihood of borrowing other used injection equipment and backloading would remain high, however, since only SSPs provide other injection equipment (e.g., cotton filters, cookers, rinse water) and harm reduction education. The lack of a statistically significant association between distance to fixed-site SSP and borrowing used syringes in the overall sample might also be explained by the possibility that distance to fixed-site SSP and borrowing used syringes are only associated among PWID who usually travel by means other than an automobile. When we stratified our analysis by means of transportation, living more than 3 miles from a fixed-site SSP (versus living within 3 miles) was strongly associated with a higher prevalence of borrowing used syringes among those who traveled by means other than an automobile but was not meaningfully associated with borrowing used syringes among those who traveled by automobile.

Interestingly, borrowing other used injection equipment was associated with living farther from a fixed-site SSP (> 3 miles vs. ≤ 3 miles) among *both* those who traveled by automobile and those who traveled by other means. It is unclear why SSP proximity would be related to borrowing other used injection equipment and not any other outcome among PWID who traveled primarily by automobile. One possible explanation is that participants were not as motivated or concerned about obtaining other clean injection equipment as they were about obtaining clean syringes. Therefore, living far from a fixed-site SSP may have been enough of a barrier to deter PWID from obtaining other injection equipment, even when an automobile was available.

Fixed-site SSPs have been frequently conceptualized as structural interventions that alter the environment in ways that could potentially decrease the risk of HCV transmission for all PWID in close physical proximity to a fixed-site SSP, not just those who directly utilize SSPs themselves. Two previous studies empirically evaluated this hypothesis by stratifying their analyses by SSP use [16, 48]. The first study, conducted in New York City, found that the negative association they observed between spatial access to SSPs and the odds of injecting with a used syringe were not stronger in magnitude among PWID who reported using an SSP in the past 6 months [16]. The second study, analyzing data from 17 U.S. cities, observed that both PWID who reported using an SSP in the past 12 months and PWID who did not were less likely to share syringes the closer they lived to the nearest SSP [48]. The authors of both studies highlighted their results as evidence that SSPs benefit PWID who live nearby, regardless of their SSP use. Without the sample size for a similar stratified analysis, we restricted our analysis to PWID who primarily obtained syringes from sources other than an SSP and observed that living a farther distance from a fixed-site SSP remained associated with a higher HCV seroprevalence and a higher prevalence of injection equipment sharing practices. We too believe that our findings lend support to the conceptualization of fixed-site SSPs as structural interventions. However, adequately testing this hypothesis would require a more rigorous causal mediation analysis than our cross-sectional sample would allow.

Our findings should be interpreted in the context of several limitations. These data are cross-sectional, meaning we cannot draw conclusions about causal relationships. As previously mentioned, we only had data on participants’ HCV antibody status and therefore could not distinguish between incident, prevalent, or resolved cases. Our analysis also did not consider participants’ spatial proximity to pharmacies, another potential syringe source. In our study area, pharmacies were permitted but not obligated to sell syringes without a prescription. Because the choice to sell syringes to PWID is often at the discretion of individual pharmacists, identifying which pharmacies offer nonprescription syringe sales is difficult and prone to misclassification. Similarly, we could not accurately account for the extent to which participants received sterile injection equipment from individuals in their social network (e.g., friends, partners, drug dealers), which could confound the study results if associated with participants’ distance to the nearest fixed-site SSP. Additionally, the fact that 4 of the 11 recruitment sites were co-located with fixed-site SSPs could have introduced selection bias. Participants recruited at an SSP are more likely to live within 1 mile of an SSP (our reference category) and more likely to be SSP clients, which could lower their risk of HCV and injection equipment sharing practices. If so, this could bias our results away from the null. This was likely mitigated, at least in part, by our use of RDS. Although the initial seeds recruited at sites co-located with SSPs were more likely to live close to an SSP and use SSP services, this was not necessarily true of the peers they recruited into the study, or the subsequent waves of participants recruited via chain-referral.

Other limitations include our small sample size, which limited our statistical power to detect associations between exposure categories and the outcomes of interest, especially in stratified analyses. The use of self-reported data creates the possibility for social desirability bias. However, participant data were collected using an audio computer-assisted survey (ACASI), which decreases socially desirable responses to sensitive questions [49]. Additionally, although informed by literature and the local geography of our study area, the cutoffs we chose for distance to the nearest fixed-site SSP are still relatively arbitrary. It is possible that different cutoffs could have better captured meaningful walking and driving distance categories for our study population. Given the relatively large geographic area of our study, it is also possible that different cutoffs would have been ideal for different regions of our study area. Finally, although we used RDS for recruitment, we did not use RDS weights in our models out of concern for introducing bias and type 1 error [47]. Therefore, our sample should be treated as a convenience rather than a representative sample, meaning there remains a risk of selection bias.

Despite these limitations, this study fills a knowledge gap regarding the impact of SSP accessibility on rural PWID. Our work also builds on the methods of previous studies by incorporating data on means of transportation, a factor especially salient in rural settings. Taken together, our findings suggest that policies that restrict the locations where fixed-site SSPs can operate (e.g., requiring local government approval, prohibiting SSPs in school zones) or create barriers to opening a fixed-site SSP (e.g., a lack of state funding, mandating SSPs provide additional health or social services) [50] may impede PWIDs’ capacity to reduce HCV-associated injection equipment sharing practices.

## Conclusion

Among a sample of PWID living in the rural northeastern U.S., PWID living farther away from the nearest fixed-site SSP had a higher HCV seroprevalence and a higher prevalence of injection equipment sharing practices. These associations were generally stronger in magnitude among PWID who usually traveled by means other than an automobile, suggesting that transportation has an important influence on how rural PWID experience distance to an SSP. Our findings suggest that the benefits of living near a fixed-site SSP may extend to PWID who do not directly utilize SSP services, although further studies are needed to evaluate this more rigorously. Overall, these findings reiterate the need for policy changes that increase the spatial accessibility of fixed-site SSPs for rural PWID.

### Supplementary Information

**Additional file 1**. **Table S1**. Associations between distance to nearest fixed-site SSP and primary and secondary outcomes, restricted to PWID who primarily obtained syringes from sources other than an SSP.

## Data Availability

All data generated and analyzed during the current study are available from the corresponding author on reasonable request.

## Abbreviations

HCV: Hepatitis C virus
PWID: People who inject drugs
SSP: Syringe services program
DISCERNNE: Drug Injection Surveillance and Care Enhancement for Rural Northern New England
NH: New Hampshire
VT: Vermont
MA: Massachusetts
RDS: Respondent-driven sampling
ACASI: Audio computer-assisted self-interview
GIS: Geographic information system
MOUD: Medications for opioid use disorder
PR: Prevalence ratio
aPR: Adjusted prevalence ratio
CI: Confidence interval
