# Supplementary material for: Association of spatial proximity to fixed-site syringe services programs with HCV serostatus and injection equipment sharing practices among people who inject drugs in rural New England, United States
Source: Harm Reduct J. 2024 Jan 28;21:23. doi: 10.1186/s12954-023-00916-5 (PMC10822149; doi:10.1186/s12954-023-00916-5)
Supplement: Supplementary file 1 — Additional file 1. Table S1. Associations between distance to nearest fixed-site SSP and primary and secondary outcomes, restricted to PWID who primarily obtained syringes from sources other than an SSP. [file 12954_2023_916_MOESM1_ESM.docx]

**Table S1.** Associations between distance to nearest fixed-site SSP and primary and secondary outcomes, restricted to PWID who primarily obtained syringes from sources other than an SSP (n = 242)

| Outcome | Distance to nearest SSP | Crude PR (95% CI) | Adjusted PR^a^ (95% CI) |
| --- | --- | --- | --- |
| HCV seropositive | ≤ 1 mile | reference | reference |
|  | 1 to 3 miles | 1.11 (0.89-1.37) | 1.15 (0.92-1.43) |
|  | 3 to 10 miles | 1.21 (1.06-1.38) | 1.21 (0.99-1.47) |
|  | > 10 miles | 1.12 (0.90-1.40) | 1.13 (0.96-1.32) |
| Borrowing used syringes | ≤ 1 mile | reference | reference |
|  | 1 to 3 miles | 0.79 (0.57-1.08) | 0.84 (0.58-1.21) |
|  | 3 to 10 miles | 0.83 (0.57-1.21) | 0.89 (0.66-1.19) |
|  | > 10 miles | 1.11 (0.64-1.95) | 1.14 (0.53-2.44) |
| Borrowing other used injection equipment | ≤ 1 mile | reference | reference |
|  | 1 to 3 miles | 0.81 (0.62-1.05) | 0.95 (0.70-1.31) |
|  | 3 to 10 miles | 1.30 (1.05-1.60) | 1.37 (1.09-1.71) |
|  | > 10 miles | 1.29 (1.03-1.62) | 1.33 (1.16-1.54) |
| Backloading | ≤ 1 mile | reference | reference |
|  | 1 to 3 miles | 0.93 (0.72-1.20) | 1.12 (0.90-1.38) |
|  | 3 to 10 miles | 1.15 (0.75-1.78) | 1.31 (1.00-1.71) |
|  | > 10 miles | 1.31 (0.53-3.20) | 1.39 (0.97-2.00) |

PR = Prevalence ratio; CI = Confidence interval  ^a^Adjusted for age, gender, race, sexual orientation, incarceration, homelessness, years of injection, injection frequency, inject multiple times per sitting, inject heroin, inject cocaine, inject methamphetamine, inject speedball/goofball, ever received medication for opioid use disorder
